# Supplementary material for: PLGA/PEG Nanoparticles Loaded with Cyclodextrin-Peganum harmala Alkaloid Complex and Ascorbic Acid with Promising Antimicrobial Activities
Source: Pharmaceutics. 2022 Jan 7;14(1):142. doi: 10.3390/pharmaceutics14010142 (PMC8780513; doi:10.3390/pharmaceutics14010142)
Supplement: Supplementary file 1 [file pharmaceutics-14-00142-s001.zip › pharmaceutics-1498751-supplementary.pdf]

pharmaceutics

MDPI

PLGA/PEG Nanoparticles Loaded with Cyclodextrin-*Peganum harmala* Alkaloid Complex and Ascorbic Acid with Promising Antimicrobial Activities

Sherif Ashraf Fahmy , Noha Khalil Mahdy , Hadeer Al Mulla , Aliaa Nabil ElMeshad , Marwa Y. Issa and Hassan Mohamed El-Said Azzazy

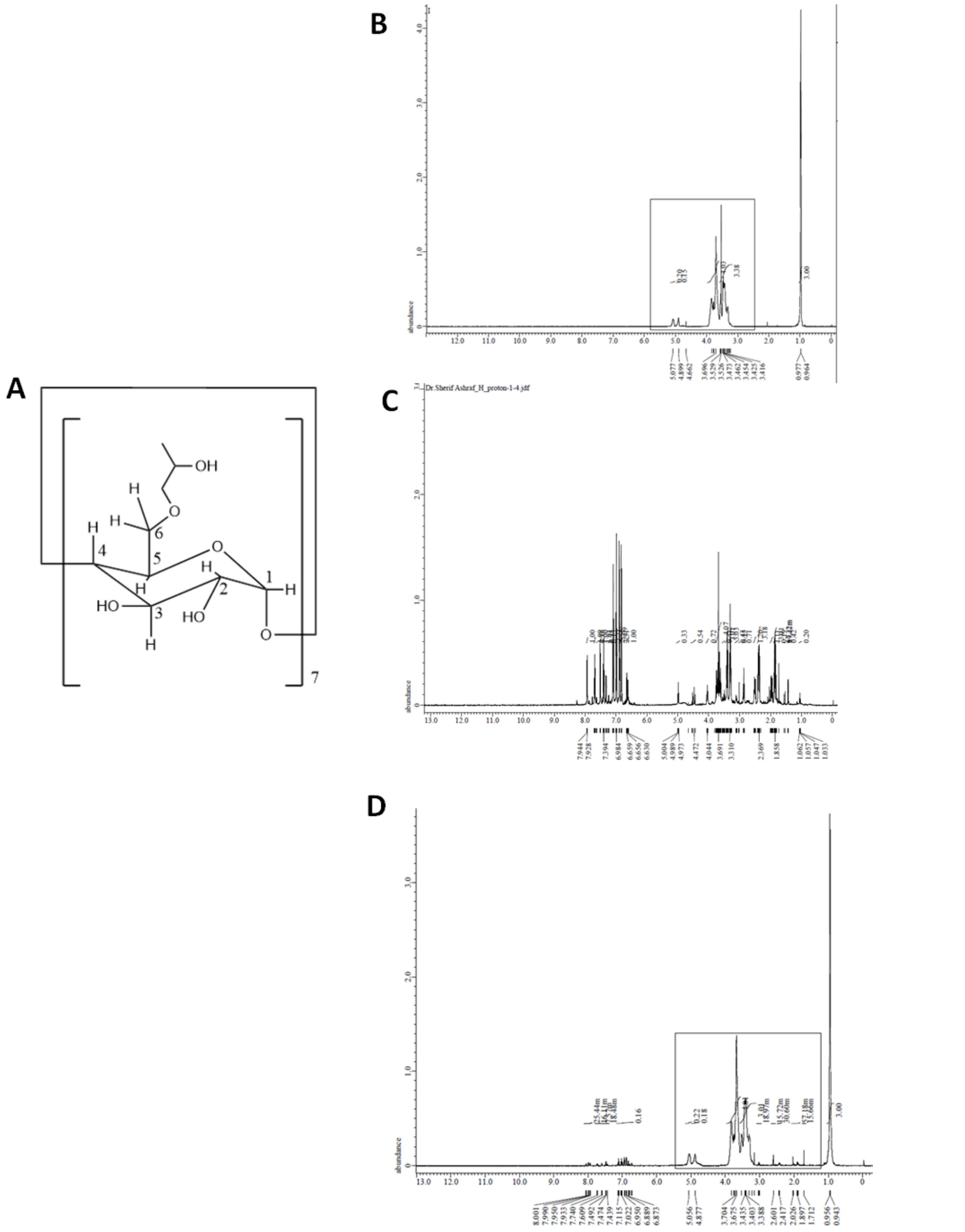

Figure S1. (A) Structure of HPßCD with appropriate protons numbered. ^{1}H NMR spectra of (B) HPßCD, (C) HARF, and (D) HARF–HPßCD complex.
